# Supplementary material for: The interactome of intact mitochondria by cross-linking mass spectrometry provides evidence for coexisting respiratory supercomplexes
Source: Mol Cell Proteomics. 2017 Dec 8;17(2):216–32. doi: 10.1074/mcp.RA117.000470 (PMC5795388; doi:10.1074/mcp.RA117.000470)
Supplement: Supplemental Data [file supp_RA117.000470_133922_0_supp_23978_fzffwf.zip › spectra_annotation/mito_spectra_annotation/405-1-13-1-5-1.pdf]

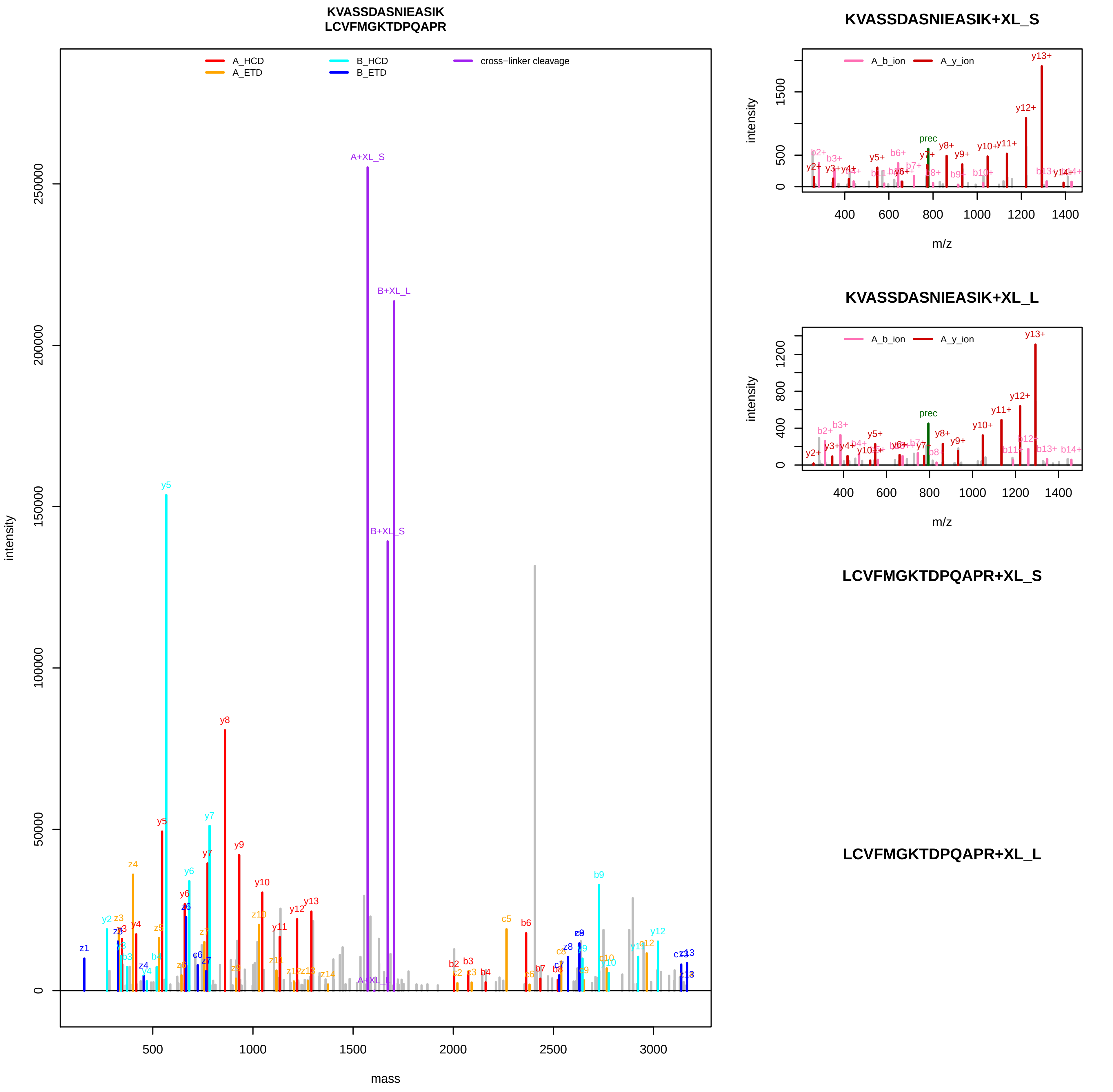
KVASSDASNIEASIK
LCVFMGKTDPQAPR
A_HCD
A_ETD
B_HCD
B_ETD
cross−linker cleavage
A+XL_S
B+XL_L
B+XL_S
y5
y8
y9
y10
y7
y6
y13
y12
y11
z4
b9
c5
b6
y12
intensity
mass
0
50000
100000
150000
200000
250000
500
1000
1500
2000
2500
3000
KVASSDASNIEASIK+XL_S
A_b_ion
A_y_ion
y13+
y12+
prec
intensity
m/z
0
500
1500
400
600
800
1000
1200
1400
KVASSDASNIEASIK+XL_L
A_b_ion
A_y_ion
y13+
y12+
y11+
y10+
prec
intensity
m/z
0
400
800
1200
400
600
800
1000
1200
1400
LCVFMGKTDPQAPR+XL_S
LCVFMGKTDPQAPR+XL_L
